# Exploring the Implementation of UNESCO’s MAB Program in South Africa: A Case Study of the Cape Winelands Biosphere Reserve

**DOI:** 10.1007/s00267-024-02048-3

**Published:** 2024-09-18

**Authors:** Michael Klaver, Bianca Currie, James G. Sekonya, Kaera Coetzer

**Affiliations:** 1 Department of Conservation Management, Nelson Mandela University, George, South Africa; 2 Sustainability Research Unit, Nelson Mandela University, George, South Africa; 3 Department of Geography, Geoinformatics and Meteorology, University of Pretoria, Pretoria, South Africa; 4 Global Change Institute, University of the Witwatersrand, Johannesburg, South Africa

**Keywords:** UNESCO, Man and the Biosphere Program, Biosphere reserve, Social-ecological system, Governance, Sustainable development goals.

## Abstract

The Man and the Biosphere Program (MAB) responds to challenges of the Anthropocene through an explicit social-ecological approach. Implemented as a world network of biosphere reserves, MAB aims to increase [eco]system sustainability and resilience globally, via individual model sites for learning and sustainable development. This research provides an in-depth case study of MAB implementation in South Africa using the Cape Winelands Biosphere Reserve (CWBR), established in 2007 when a key MAB guiding policy, the Madrid Action Plan came into effect. The study utilized semi-structured in-depth interviews with strategic and operational management, and document analysis. The CWBR prioritizes their role as a landscape coordinator, a driver of socio-economic development and site in which humans derive benefits from healthy natural environments. The CWBR have adopted a non-profit organization cooperative governance model in support of this vision, fulfilling the socio-economic development function primarily through successful international partnerships. Challenges faced include a perceived lack of sufficient government support, limited stakeholder awareness and insufficient resources for project implementation. Over reliance on the pillar of their model, the chief executive officer in the current governance form, is an instrument in their effectiveness, yet carries significant risk. These are learnings useful for other biosphere reserves translating an international designation for a local context.

## Introduction

The United Nations Educational, Scientific, and Cultural Organization (UNESCO) Man and the Biosphere Program (MAB) is implemented through biosphere reserves (BRs) which are holistic social-ecological system (SES) management tools serving three functions, including conservation, sustainable socio-economic development and logistic support, i.e. education, research and monitoring (UNESCO 1996). They involve inclusive, integrated, flexible and multistakeholder governance arrangements that are context specific and useful in dealing with the interlinked triple challenge of the Anthropocene, i.e. climate change, biodiversity loss and human wellbeing in a growing population (Pool-Stanvliet 2013; Palomo et al. 2014; Carruthers 2020; Baldwin-Cantello et al. 2023). These governance approaches offer both knowledge and functional diversity (Müller 2014). Moreover, BRs are regarded as sustainability science and climate change learning sites (UNESCO 2007; Pool-Stanvliet 2013; Pool-Stanvliet and Coetzer 2020; Clüsener et al. 2022; Barraclough et al. 2023) and when considered as a world network they provide a platform to share innovation and best practice globally. Additionally, the place-based implementation of MAB is flexible, meaning BRs can be adaptive, and factors of redundancy and modularity can be incorporated within their governance arrangements—all factors increasing system resilience (Müller 2014).

To improve the effectiveness of BR governance globally UNESCO policy recommends several structural components. The Technical Guidelines for Biosphere Reserves (UNESCO 2021), recommend a dedicated management team, steering or executive committee and advisory board. The Lima Action Plan (LAP) 2016–2025 (UNESCO 2017) calls for joined alliances for open participation and planning, as appropriate models contributing to the implementation of Sustainable Development Goals (SDGs).

In a systematic literature review of BR management effectiveness (66 publications between 1996 and 2017), Ferreira et al. (2020) found 57,6% research emanated from the Global North and very few (6%) publications were in-depth case studies on BR management and governance—of which most were project-based. The outcome of the review was a need for greater geographic diversity and in-depth single case studies of BRs in research. Barraclough et al. (2023) found BRs remain underutilized research contributors to sustainability science theory and practice. Further, literature on BRs has identified the need for a place-based understanding of the institutional context and governance strategies of BRs (Coetzer et al. 2014; Ferreira et al. 2018; Pool-Stanvliet and Coetzer 2020; Barraclough et al. 2023). Currently, there is a drive *“to communicate the experiences and lessons learned, facilitating the global diffusion and application of these models”* (UNESCO 2017:15). Therefore, understanding how MAB is implemented across BR sites and comparing lessons learnt is needed to improve implementation success (Coetzer et al. 2014; Klaver et al. 2024).

To address the need for greater geographic diversity, in-depth case studies and understanding the institutional context and governance strategies of BRs, South African scholars are currently conducting research in South African BRs (more can be found here https://researchbiosphere.org, accessed on 4 June 2024). The goal is to compile in-depth case studies of South African BRs to uncover aspects of MAB implementation, i.e. lessons and experiences learned, and to share these across the World Network of BRs (WNBR) in the hopes of improving MAB implementation success (see Klaver et al. 2024). A strength of MAB is the lack of prescriptions for implementation—an intentional avenue for learning—however, how global policy is contextualized for local application is not well understood. Therefore, exploring how MAB is implemented in various contexts is important and remains a gap. The ‘one size fits all’ approach does not comply with the BR model which necessitates learning-by-doing and sharing these experiences across the world network of BRs (Ishwaran et al. 2012; Pool-Stanvliet and Coetzer 2020) specifically for understanding what works, why and in which contexts.

This case study aims to provide a contextual understanding of MAB implementation in local conditions, and not to produce findings which are generalizable. Rather, we aim to share lessons and experiences learned through MAB’s place-based implementation across the WNBR. The Cape Winelands Biosphere Reserve (CWBR) case study may offer valuable lessons for the WNBR as it has passed through multiple generations of MAB action plans, from the Madrid Action Plan (MAP) 2008–2013 to the LAP. Therefore, the CWBR should idealy be adaptive in its approach to ensure continued alignment with the UNESCO-MAB strategic policies, for example MAP priorities of climate change, ecosystem services and urbanization (UNESCO 2008) to LAP priority of addressing the SDGs (UNESCO 2017). The CWBR is an important site within the Cape Floristic Region (CFR) World Heritage Site for its locally protected environments and catchment areas (Le Maitre et al. 2019; Department of Forestry, Fisheries and the Environment 2022; UNESCO 2022). This research explores the CWBR context of interpreting and implementing MAB and focusses on how the international designation is translated for the local social-ecological and economic fit, including the strategic decisions and motivations which support the BR in meeting its envisioned role in the landscape. In doing so, we hope to answer the question: how has the governance strategy and structure of CWBR helped achieve a context appropriate interpretation and actioning of MAB in the landscape?

## Methods

### Study Area

**Fig. 1:**
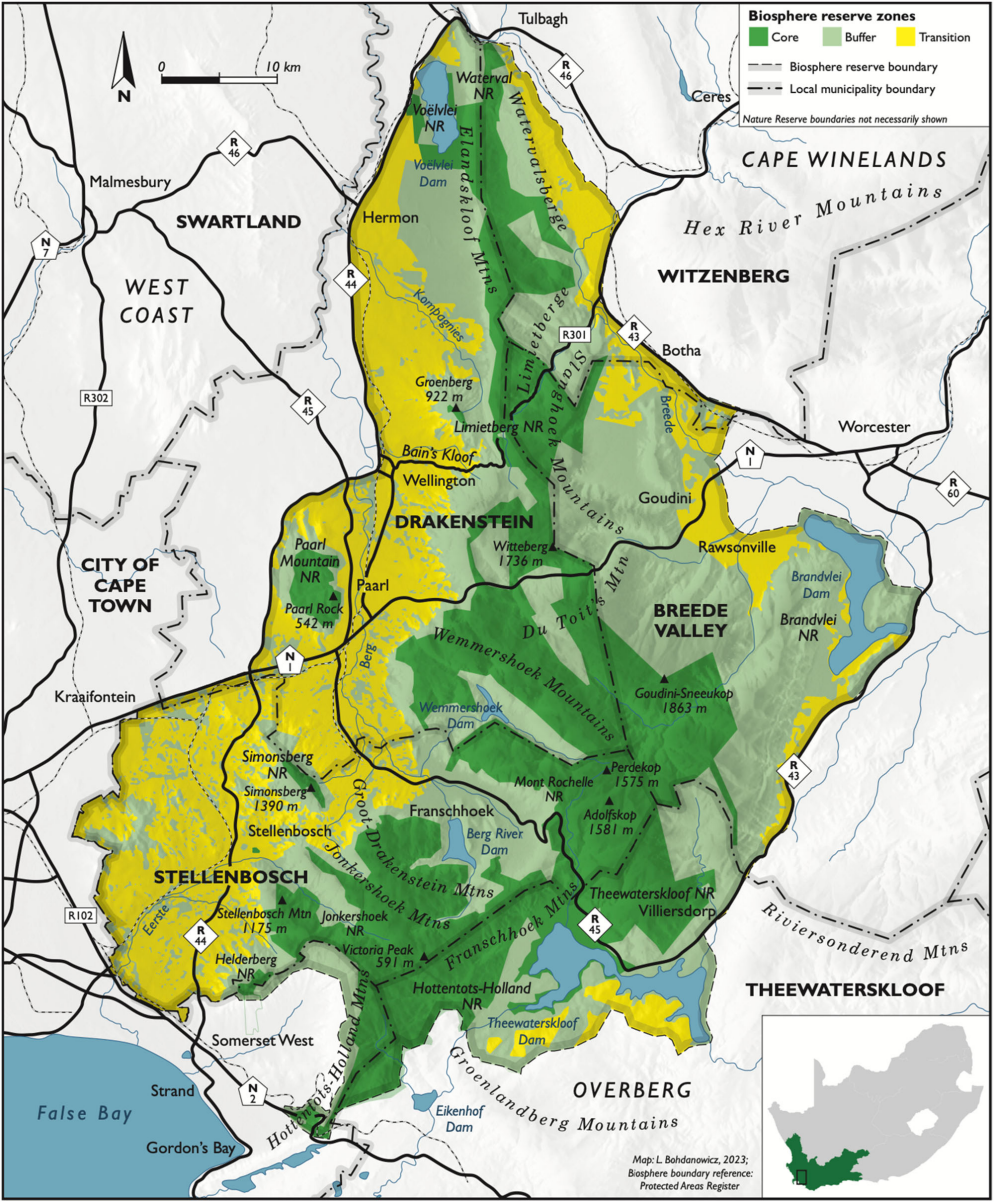
Map of the Cape Winelands Biosphere Reserve illustrating the core (30%, 99 459 ha), buffer (42%, 133 844 ha) and transition (28%, 88 727 ha) zones, important points of interest, and major towns and transport routes (Bohdanowicz 2023)

The CWBR (Fig. 1), located 40 kilometers east of Cape Town, South Africa was established in 2007, and spans roughly 322 030 ha (UNESCO^ 2022^). Furthermore, the BR lies within the CFR, which exhibits high levels of endemism and is home to 20% of the total number of plant species which occur on the African continent (Department of Forestry, Fisheries and the Environment^ 2022^; UNESCO 2022). The site constitutes a mosaic of diverse ecosystems and landscape features within the Cape Fold Mountains and surrounding valleys, displaying a variety of land-uses, including historic towns, a world-famous viticultural landscape, and some of the CFRs most important protected environments and river systems which feed the City of Cape Town (CoCT) (Le Maitre et al. 2019; Department of Forestry, Fisheries and the Environment 2022; UNESCO 2022).

The core zone of the CWBR constitutes 30% of the BR (99 459 ha) and is made up of formally protected areas managed by CapeNature^1^. The core zone prioritizes nature conservation and long-term protection (Schultz et al. 2020; UNESCO 2021). The buffer zone constitutes 42% of the BR (133 844 ha) where emphasis is placed on scientific research, monitoring, and education with limited human use and which is compatible with conservation objectives (Schultz et al. 2020; UNESCO 2021). The transition zone constitutes 28% of the BR (88 727 ha) and where human populations strive for sustainable resource management and development (Schultz et al. 2020; UNESCO 2021).

The CWBR overlaps with several municipalities including, the entire Stellenbosch Municipality, majority of the Drakenstein Municipality and sections of the Breede Valley, Theewaterskloof and Witzenberg municipalities. Furthermore, this region has a winemaking tradition and history dating back approximately 350 years, and accounts for 68% of the total wine production within South Africa (UNESCO 2022). Other primary economic activities in the area include agriculture, manufacturing, tourism, forestry, business services and real estate (Department of Forestry, Fisheries and the Environment 2022).

### Methods and Data

This case study was conducted following a qualitative approach (ethics certificate: H22-SCI-NRM-001), using semi-structured interviews (*n* = 8) and supplemented through document analysis (*n* = 3). Criterion for participant selection were that they represented the current CWBR strategic and operational management personnel, deemed by the research team as the most relevant operationalization of the governance strategy and structure of the CWBR at the time of the research. Therefore, the Board of Directors (BofD) and executive management (chief executive officer and coordinator) were purposefully sampled with a response rate of 88.89% (*n* = 1 participant unavailable for an interview).

Interview transcriptions (and documents) were thematically coded manually by the primary author of this paper using ATLAS.ti (2022) 23.2.0 for Mac and analyzed through the inductive 6-step thematic analysis described by Braun and Clarke (2006) following respondent validation. Thematic analysis is an inductive qualitative data analysis method used to identify, analyze and report on repeated patterns, i.e. ‘themes’, observed in the data to provide an in-depth understanding (Braun and Clarke 2006; Humble and Mozelius 2022). The process included transcribing the data, coding interesting features across the entire data set, collating codes into potential themes, reviewing fit of themes in relation to quoted extracts, refining and describing themes, and thereafter, reporting findings.

Frequency (*f*) counts within the results refer to the number of participants mentioning a certain theme and are used to indicate the significance and not for quantitative analysis purposes. Verbatim quotes have been used where possible, however in some cases minor alterations were needed to enhance the ‘readability’ of the data, i.e. repetitions, hesitations, stumbling speech, or translations have been removed (Brennan 2022). Additions for clarification and redactions have been made in the form of [XXX] or ‘…’ to protect identities. Quotations have been used in-text or provided in quotation tables. Source codes for all quotations, verbatim and those incorporated into the narrative structure of the results, were removed to enhance readability (Lingard 2019). All quotations are attributed anonymously in Supplmentary Table 1, providing the necessary audit trail for validation of research results.

#### Semi-structured Interviews

The following overarching themes were explored through the interview protocol:

- **Governance model**: How the governance model of the BR was initially formed, how this enables a specific governance approach, i.e. how decision-making processes occur, and how it has changed over the years.
- **History of involvement**: Sources of personal commitment to the BR and the motivations for the participant’s initial and continued involvement in the BR.
- **Envisioned role**: Perceived vision for the BR, and the BRs role in the SES.
- **Stakeholders**: How the current BR governance model allows, enables/prevents other stakeholders from participating in the BR and how participation is constructed by the BR.
- **Critical relationships**: Knowing the institutional overlap in the landscape the BRs functions within, as well as the horizontal and vertical alignment of actors operating in the landscape.

Interviews were designed to be informal discussions. Interviews ranged from 45 to 130 miutes and were conducted in May 2023 at the interviewee’s location and time of choosing (two interviews were conducted virtually). Within each of the overarching themes there were several sub-questions used to facilitate the discussions and probe for more detail, clarification or to change direction of the interview.

#### Document Analysis

Document analysis involved the use of the CWBRs website (https://www.capewinelandsbiosphere.co.za), the BR-specific spatial development plan and staff and stakeholder spreadsheet provided by the CWBR.

## Results

### Governance Model: Initial Establishment and Evolution

The CWBR was initiated by the Cape Winelands District Municipality (CWDM) and the proposal put together by the Dennis Moss Partnership – a top-down establishment. The CWBR was registered in 2007 after a four year long public participation process. The impetus for its establishment was that it would be an important spatial development planning instrument and that development proposals would have to be approved by the committee. This function is fulfilled by the Department of Environmental Affairs and Development Planning, so to avoid duplication this role was never realized. The CWBR was initially well received by its stakeholders; however, expectations did not meet reality and *‘damage control’* was needed for the first two years—seeing to the promises made by the consultants writing up the proposal.

The role of the CWBR has evolved since its establishment. Their initial strategy was to focus on one BR function at a time, build momentum and then focus on the next. The CWBR *“didn’t try to deal with all three* [BR core functions] *at a time. So* [the CWBR] *concentrate*[d] *on the education side”*. With the belief that education is the answer to many current issues, their initial foci was conservation and education. They formed partnerships with European funders, thereafter *“*[the CWBRs] *focus has been more community upliftment and education based*”. *“The next side* [the CWBR] *were weak, was the scientific side”*, and recently the CWBR has become more involved in science and research. The CWBRs projects are intended to align with the core functions of BRs as stipulated in The Statutory Framework of the World Network while responding to emergent local challenges.

### Governance Model: Structure, Responsibilities and Decision-Making

**Fig. 2:**
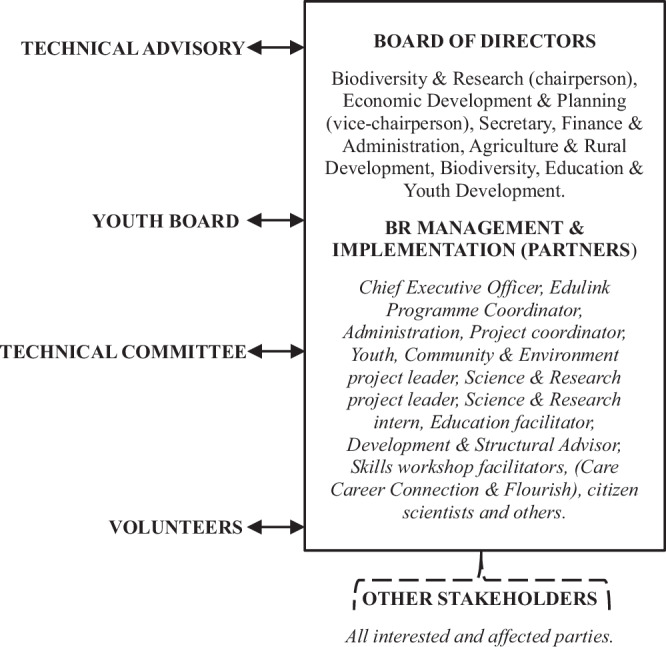
Schematic diagram of the Cape Winelands Biosphere Reserve personnel, including its technical assistance, board of directors, management and implementation team, youth board, volunteers and other stakeholders

Leading the CWBR are the BofD. The management team, including executive management, project leaders and others oversee implementation within the CWBR. The BofD, management team, technical advisory and youth board make up the management entity, which is the CWBR, a Section 21 non-profit company^2^ (Fig. 2). Supporting them is the technical committee and a well-established volunteer program.

The board consists of seven directors (1 female, 6 male) with a chairperson, vice-chairperson, secretary, and several portfolios. In terms of the diversity, a participant believes *“getting gender and racial representation on the board remains a challenge”*. Each director is assigned to a portfolio, depending on their expertise, or a portfolio may be established for them. The responsibility of the BofD is to provide an operational oversight role. Linkages are present between the directors and some of the stakeholder groups to allow information to be shared and for fostering collaboration, for example with the CWDM, Heritage Committee, Iziko Museums, CapeNature and universities. The *“board’s role*, [to] *a large extent, is to communicate and have that relationship with stakeholders”*.

CWBR management consists of a chief executive officer (CEO), administrator and coordinator, and several project leaders, advisors or facilitators. The CEO leads the management, oversees implementation, builds networks and relationships with funders and partners, and is tasked with timely decision-making. The responsibility of the coordinator is reporting, managing records and administrative tasks. Project leaders are appointed through service-level agreements and work part-time managing projects and facilitating various activities.

Directors are either sourced by or approach the CWBR to be elected to serve on the board. Personnel are sourced for their interest, skills and knowledge, and networks (Table 1). Other criteria may include whether they are involved in activities aligning with the BR objectives, and although not important at present, reside within the CWBR. Currently, *“the main thing is, what skills do* [the CWBR] *need?”*. Directors are retained until they are unwilling to serve or the lack of alignment between the objectives of the CWBR and what the director provides. Whilst there are recent additions to the BofD, i.e. within the last 2 years, most of the directors have served for a minimum of five to seven years, with some (*n* = 3) serving since the establishment of the CWBR, including the CEO. The CWBR has had a full-time coordinator since 2018.

**Table 1:** Descriptions of beneficial skills within the Cape Winelands Biosphere Reserve office helping to fulfill its envisioned role

Skill	Skill Description
Personal	Personnel bring the people skills and the ability of *‘connecting with people’*, the *‘collaborative spirit’* and *‘leadership skills’*, technical *‘writing skills’* and *‘objectivity’*.
Knowledge	*‘Built environment’*, specifically the interface between the built and natural environments, *‘environmental* and *conservation ecology’* knowledge, as well as *‘business’*, *‘town and regional planning’*, and *‘sustainable development’*.
Networks & connections	Spheres within government, public institutions, and the field of conservation.

The CWBR has four full-time staff, including the CEO, coordinator, and project leaders whose work hours are flexible. These staff have specific responsibilities or commitments which need to be delivered upon. This generally entails a 40-hour week but can be much longer – depending on what is required. There are five to eight part-time workers depending on project funding, including administration, project leaders, advisors and facilitators. Opportunities for training exist, both formal and informal, for example the coordinator completed a virtual BR management course. Participants believe important full-time positions for the CWBR that should be filled include a CEO, coordinator, administrator, project leaders and a social media manager.

Board meetings, held quarterly, are a place for the BofD and CEO to plan, discuss and vote on decisions. Two thirds (4/6) of the BofD must be present for board meetings, including the secretary, chairperson and CEO, and all present need to vote. Decision-making is perceived to be an informal process and *“the board meetings are a space to have conversations about things—to give input. But I wouldn’t say there’s a formalized decision-making structure … I think it’s quite loose”*. Meeting agendas are shared beforehand allowing items to be attached. Ad hoc meetings occur regularly, for example with municipalities—allowing decisions to be made quickly. The CWBR has what is called an *‘open phone policy’*—meaning the CEO calls on individual directors depending on which portfolio expertise are needed to make decisions quickly. There is trust between the CEO and directors and often decisions are made independently of the BofD and discussed at a later stage. The CEO leads CWBR management meetings, a place to provide updates and work through programs, on a weekly basis. In these meetings there are usually 14–22 individuals depending on absentee numbers.

With regards to decision-making around CWBR projects, decisions are not made without the local communities. CWBR *“spend of lot of time in the communities”* attending community meetings through invitation where there is open dialog in *‘co-creating’* projects and solutions. Through these relationships being built, communities can regard the CWBR as a channel to the municipalities when they cannot get answers themselves, specifically regarding issues of housing, education and access to land.

There are no memberships, however the CWBR has a volunteer program. There are generally six to eight volunteers (maximum of 14) who are either local or international. Volunteers help with ongoing projects or initiate new projects depending on their skill set, for example the *‘drone project’*. The CEO often meets with the prospective volunteers beforehand in attempt to match the volunteers with specific projects or needs. It is clear that *“BRs couldn’t run as well as they do without volunteers and people being able to give of themselves”*—relying on many voluntary hours and the goodwill of people, including the BofD.

CWBR personnel have similar motivations for joining and continuing their service, including their personal interest (*f* = 6), enthusiasm and enjoyment, and appreciating the holistic and inclusive approach of BRs (*f* = 5), being able to give back to the community (*f* = 4), the dynamics of what the role entails (*f* = 3), prospects of personal growth and opportunities (*f* = 3), believing that these are good people doing good work (*f* = 2) in their local environment, and caring for the environment (*f* = 2).

The CWBR has a linked youth board (est. 2021), situated outside the primary board which supports project implementation. The youth board consists of youth from different local communities and is regarded as a *“very powerful way of embedding* [the CWBR] *in communities”*. It has been an experimental process and seen by some as relatively unsuccessful and therefore to be evolved into a youth committee or forum.

Supporting the effectiveness of the CWBR is the technical advisory and technical committee. The advisory consists of individuals with knowledge on sustainable living and UNESCO-MAB. The committee, which fulfills an advisory role to the board, consists of critical stakeholders, for example the local municipalities, CWDM, Department of Agriculture and CapeNature. The committee meet once or twice a year and at the annual general meeting (AGM) to share knowledge and information, and feedback on activities. A participant summarizes the experience below:

“It’s almost like stakeholder involvement… these are all the various stakeholders that are interested in the CWBR, and they need to be represented … on the technical committee to make sure that they are happy with the direction [the CWBR] is going. …, most of the time the technical committee is made up of people that are coming from organizations that are already doing things. It should almost be for them to come along and tell us what they’re doing. Then we can find out where we can help them a bit more, which does happen. But generally, those meetings, there’s a lot of very quiet people there.”

### Envisioned Role: Present Role in the Landscape

The CWBRs current role within the larger SES is grouped into three themes, including socio-economic development, collaborating with, and coordinating actors in the landscape, and conservation. The CWBRs **collaboration and coordination** role is to *‘support and facilitate’* actors in the landscape. Participants perceive the CWBR as an organization to network, connect actors and to help those in need. This enables the CWBR to partner with relevant actors when problems arise. A participant stated, *“*[the CWBR] *will look at the problem. Diagnose it. What partners do* [the CWBR] *need to sort that out”*.

**Socio-economic development** has been their focus because of the disparity in education in South Africa and the need to understand issues to solve them. Their educational programs have been successful and gained them support. There is an attempt to *‘align with government departments’* and *‘fill the gaps’* with their projects, for example early childhood development (ECD) a niche of the CWBR. Their ECD program has put teachers through training and offered learning experiences with Iziko Museums. The CWBR offer other forms of training for all interested parties, for example woodwork to upskill the local communities. The CWBR conducts environmental education and outreach programs with the youth and uses a mobile science unit (trailer) to promote conservation at schools.

The CWBRs **conservation** role is fulfilled through partnerships with several organizations, for example invasive alien plant (IAP) clearing with WWF and Idas Valley Trails, and with universities to fulfill their role in science and research. The CWBR have developed strategic partnerships with several universities including, University of Stellenbosch, University of Cape Town—specifically their African Climate Development Initiative, University of Leuven, and the Flemish Institute for Technological Research in Belgium. The CWBR support provincial government, WWF and CapeNature in research and monitoring through baseline data collection with the use of drones—a project initiated by a volunteer. There are citizen science projects, for example their Source-to-Sea BeResilient project which focuses on conserving rivers and environmental education.

### Envisioned Role: Challenges

Three themes emerged as challenges for the CWBR to fulfill its envisioned role, including the lack of resources (Table 2), limited support from national government (Table 3), and community stakeholders who are unaware of the CWBR or dealing with other social issues (Table 4). These challenges are discussed further below.

**Table 2:** Resources as a challenge for the Cape Winelands Biosphere Reserve in fulfilling their envisioned role

Theme	Example quotes
Lack of human & financial resources	*“Funding is a major challenge. Most of the guys are doing it for the love of conservation.”* *“*[The CWBR are] *still very reliant, heavily reliant on* [the CEO] *and* [the coordinator]*. There’s a need to invest in human resources for the organization. Which comes along with funding questions.”* *“There’s scope for more, but it requires a larger dedicated team and operational funding.”* *“I don’t understand why the Western Cape* [BR] *Forum doesn’t get together more. You know because the power of collaboration. I know it from business. I mean, there is big funding out there, but it’s too big for one biosphere, but all five biospheres. We can go for the 50* [or] *100-million-europroject. But working in silos, we can’t do that.”*

**Table 3:** Government as a challenge for the Cape Winelands Biosphere Reserve in fulfilling their envisioned role

Theme	Example quotes
Limited government support	*“It’s frustrating that our system, our top-down structure – we don’t have leadership on top.”* *“Again, who does the CWBR report to? What is our relationship? What are they doing? What is our contribution to helping them achieve their objective? Now for me that’s not defined at all. We don’t see any real government, either of provincial or national, participation really in terms of what to do. There’s got to be a chain of command in all these organizations. Who are you? What are you doing? Why are you doing it? Who’s effectively in charge of the whole thing? Power is what, we have no power. Things are devolved down to us to do, and to achieve what? To me those are not well defined, properly defined that people can understand or support for that matter. We have no support … in real terms from national or provincial government.”* *“Treasury doesn’t want money to come to the Western Cape. I mean, I’ve had that from* [XXX].*”* *“*[During technical committee meetings the CWBR] *get a ‘representative’ from the organization coming along. But not necessarily the right representative. It gets delegated down till somebody gets sent to you. That’s not always useful.”* *“I know they had challenges with the legislation … they couldn’t even transfer funds. I think 10,000 rand for reports. We compiled the report, a ten-year report. They said that that they will fund it, … they couldn’t fund it. They don’t have a mechanism of transferring funds…”* *“Guys at local government, if they have certain KPIs* [key performance indicators]*, they focus on that. If the CWBR invites them, they just say, no, I’m not available… the person, individual at that organization must have similar interests to get them in.”*

**Table 4:** Awareness and communities as a challenge for the Cape Winelands Biosphere Reserve in fulfilling their envisioned role

Theme	Example quotes
Awareness & communities	*“It’s not really well known to the public, what we do, that we are there and we do all these things. I mean, the general public is not aware of us. Maybe in Franschhoek, with other projects there. People would know about it. In Stellenbosch, if you say BR. They say, what is that?”* *“Getting communities to understand science, and climate change, and conservation is not easy.… hungry stomachs have no ears.”* *“We’ve got a difficult biosphere in that a lot of the community is gang controlled.”* *“It’s very, very difficult to get to the kids, which I think is probably one of the most important things. Especially the way things are going now. If you don’t have any understanding of the natural world, you’re going to have absolutely no desire to do anything about it.”*

**Lack of resources (*f*** = **7)** is associated with the lack of funding, specifically operational funding which influences human resources and capacity. The CWBR is believed to be over reliant on few human resources. Partnering with other BRs to source funding seems to be an underutilized strategy.

**Limited government support (*f*** = **4)** uncovers a perceived lack of MAB governance within South Africa, and therefore believed to be a lack of clarity in defined roles, structures and responsibilities within the *‘chain of command’*, i.e. national MAB governance down to CWBR. Participants believe national government are hesitant in allocating resources to the Western Cape for what is perceived to be political reasons^3^. There is the perception that their engagement is superficial. It is believed there are structural challenges with regards to government involvement, for example, limited mechanisms to transfer funds, and believing that interacting with BRs is not part of their functional responsibility, i.e. no mandated engagements.

**Awareness and communities (*f*** = **3)** is associated with the awareness of the CWBR and the concept of BRs, getting people to understand issues which are not their immediate priority, as well as challenging community dynamics due the presence of gangsterism. Participants believe the current schooling system does not provide for many extracurricular activities which makes it challenging for the CWBR to engage with the youth.

### Envisioned Role: Effectiveness

There are two factors which have led to the CWBR success, including leadership and the quality of staff. Participants suggested it is the continued perseverance and voluntary commitment of the team. Other factors include the leadership, like-mindedness, personalities and networks within the team involved. Participants attributed much of the success of the CWBR to the CEO. One participant stated: *“If it wasn’t for* [the CEO]*, none of this would have happened. None of it would happen*. [The CEO is] *a rather phenomenal person”*. Some credited the partnership between the CEO and coordinator together with the relaxed and flexible nature of engaging with the team. When asked about instrumental positions which have increased the CWBRs effectiveness, one participant believes success has come from the team’s passion and commitment to meaningful work, while another explained that it is more the types of people and personalities involved and not necessarily their position (see below).

“The structure has got nothing to do with it. If you don’t have the right people there. Or if you do have the right people, you can have any structure, the structure isn’t the issue. It’s the personalities and whether they [are] prepared to do what they’re supposed to do or not, you know. You can have the best structure in the world and a bunch of ‘palookas’ sitting in it and it’s still not going to work.”

### Stakeholders and Critical Relationships

Stakeholder participation occurs *“on a case-by-case basis”* and could include any actors in the CWBR of which CWBR stakeholder mapping has identified for example provincial government departments, non-governmental organizations (NGOs), community forums and water catchment area working groups, local businesses, community organizations and sports clubs. One participant believes partnerships cannot be forced and that one must attract them by providing value, which the CWBR have done in their educational domain. Participation from all interested and affected parties is encouraged through blog posts on their website, newsletters, word-of-mouth, and via phone call. Participation levels are increasing – several participants said that they have had increasing attendance at their AGMs.

### Stakeholders and Critical Relationships: Participation Challenges

Participants reported challenges with regards to stakeholder participation from local government: there is superficial engagement and lack of perceived significance of the CWBR. Furthermore, some stakeholders are perceived to be prioritizing their own agendas, or that the CWBR does not feature on the stakeholder’s agenda, or within their professional *‘functional responsibility’* unless the individual has a personal interest.

Stakeholder participation from local and national **government (*f*** = **4)** is believed to be missing despite being considered critical partners. Participants believed that municipalities are disinclined to play an active role in CWBR engagement/participation, with room for more engagement. Furthermore, participation from some key **conservation bodies (*f*** = **2)** are believed to be missing. The absence of these stakeholders is a *‘limitation’* for the CWBR. The CWBR would like more participation from the **public (*f*** = **2)** particularly youth groups.

### Stakeholders and Critical Relationships: Institutional Overlap and Disruptive Stakeholders

Institutional overlap in terms of mandate occurs with other actors in the landscape, for example CapeNature and municipalities. Participants believe the challenge therein is to reduce duplication, and resource competition. Participants mentioned the opportunity is to form partnerships, pool resources and collaborate – *‘dovetailing’* projects with CapeNature, for example. Participants were unsure whether such overlap enables or constrains ongoing/further government support. Participants believe it depends on who is involved as one needs a shared vision and the *‘collaborative spirit’*, while another said that it could enable support, however staff turnover is a challenge when it comes to building long-standing relationships.

Participants perceive some municipal councilors to be disruptive in their attempts to politicize the CWBR and that even directors have the potential to be disruptive by trying to push their agendas and in some way *‘hijack’* the CWBR. Furthermore, some government departments are believed to be disruptive and of little help to the CWBR – in some cases perpetuating and exacerbating issues, for example land invasions.

### Lessons Learned to Share Across the World Network of Biosphere Reserves

When asked what lessons the CWBR would share with emerging or newly established BRs, two themes emerged (Table 5), including the **type of people (*f*** = **6)** involved. Participants believe it is necessary to *“get a nice core team together”* and a good CEO or leader. There were also lessons around **BR infrastructure (*f*** = **4)**, for example a physical space is beneficial to have, setting a clear plan (strategy or business plan) early on, to find a niche, and if there are overlaps with other actors look to build partnerships and collaborate. Partnerships and continuity of funding are considered the *‘lifeline’* of a BR. A participant advised to try form these early and develop the BR around the personalities involved.

**Table 5:** Lessons learned by the Cape Winelands Biosphere Reserve to be shared across the World Network of Biosphere Reserves

Theme	Theme description	Example quotes
Type of people	Participants referring to the directors & executive management	*“Some active individuals who are enthusiastic and make their time available to their own societies… I think the important thing is to identify two or three people who are really committed and prepared to put in time.”* *“Get yourself a good CEO.”* *“You need a charismatic person that’s willing to take on the overall role. Then for him or her using your own personal charisma to persuade other people to join this enterprise.”*
BR infrastructure	Funding, partnerships, niche, physical space, & structure	*“I think having the* [physical] *space* [headquarters]*, where volunteers … can stay. There’s an office … Everything is in one place… and I think that’s valuable… it’s really the ideal scenario. You’ve got someone who is setting up and championing something like this. If they have … the physical space that they’re working in, that can become the HQ… It just makes things a lot easier.”* *“Find your niche, …* [where] *you can have an impact. It’s about just searching for that, networking, understanding the landscape.”* *“I think having … stability of funding in place, I think there’s something linked to that around kind of international partnerships and relationships. I think that’s something which* [the CEO] *has done quite well. It’s really been a lifeline to the organization, in many ways. I think … for BRs in the Global South, establishing partnerships and relationships with either funders and/or other BRs in the* [Global] *North as a way of trying to kind of create some sense of financial stability.”* *“… the* [BR] *is dependent on funding and the personalities that drive it. If the personalities that drives the BR, if they do not conform to a rigid system, a rigid structure. If it’s easier for them to implement without that structure, then I just think it should go with the flow. It shouldn’t be overregulated in terms of* [a] *set structure.”*

## Discussion

### Envisioned role of the Cape Winelands Biosphere Reserve and their alignment with UNESCO policy

The vision of the CWBR is *“to achieve an exemplary connection between people and nature, in a secured comfortable and sustainable living environment”* (CWBR 2022) and being a model site as envisioned for BRs (Clüsener et al. 2022). The findings suggest that the CWBR’s internal vision is for greater recognition and coordination across the landscape to fulfill their role in *‘collaboration and coordination’*, operating as a ‘relational hub’ (Cockburn et al. 2020). These hubs actively develop human-human, i.e. collaboration, and human-nature connections, i.e. stewardship (Cockburn et al. 2020). This role is envisioned for other BRs in South Africa, for example Kogelberg (KBR) (Klaver et al. 2024) and the Kruger to Canyons BR who perform as a *‘collaborative platform’* (Schultz et al. 2018). The CWBR prioritizes healthy natural environments in which people can connect with and derive benefits from. Furthermore, to ensure the sustainability of the BR through overcoming resource use challenges.

To achieve their vision the CWBR do not use the MAB guidelines as prescriptions in a top-down manner, but rather drive their implementation to deal with emergent local pressures identified by the local population, that broadly supports the goals for a BR, i.e. work within the framework of the three core functions of a BR (UNESCO 1996). Their approach to fulfilling the BR functions is to focus systematically on one function at a time. This provides a good lesson for dealing with limited resources but can also increase social acceptance and support by delivering positive outcomes (Lockwood et al. 2010). The support the CWBR receive from their partners is believed to be a testament of their success.

### Governance model and structure adopted by the Cape Winelands Biosphere Reserve

The CWBR implementation is structured around what the Technical Guidelines for BRs (UNESCO 2021) refers to as the ‘NGO Model’. This model is uncommon in Africa yet prevalent amongst BRs in South Africa (UNESCO 2015; Department of Environmental Affairs 2016; Hedden-Dunkhorst and Schmitt 2020). Key UNESCO-MAB policy identifies that the NGO model allows for more effective action across zones whereas the ‘authority model’ provides better “control” of the core (UNESCO 2015). Elsewhere in Africa, BRs are also implemented in a top-down fashion through the authority model (UNESCO 2015; Hedden-Dunkhorst and Schmitt 2020) which is dependent on government ministries who are primarily responsible for conservation in core zones (UNESCO 2015; UNESCO 2021). This prioritization may translate to managing BRs as traditional protected areas without consideration for buffer and trasition zones – possible symptoms of the pre-Seville Strategy designation (Coetzer et al. 2014; Van Cuong et al. 2017).

In South Africa, however, despite being nominated by national government (Pool-Stanvliet 2014), BRs have mostly been designated through bottom-up processes (Pool-Stanvliet and Coetzer 2019) and are independent, apolitical non-profit organizations (NPO) (Department of Environmental Affairs 2016) which does provide BRs with legal existence (Borsdorf et al. 2014). Core areas are protected by public authorities, in this case CapeNature, while other institutions operating in the buffer and transition zones all have their own leadership structures. In terms of legislation, South African BRs are not regulated by law and their management plans are not legally binding, however the three core functions are recognized and reflected in the country’s legislation (Department of Environmental Affairs 2016) with additional regulation of the NPOs by the Companies Act (Act 71 of 2008) (Republic of South Africa 2008). This is similar to, for example, Swedish BRs (Elbakidze et al. 2013), Fontainebleau-Gâtinais BR in France (Borsdorf et al. 2014) and the Dana BR in Jordan (UNESCO 2021). In the context of the CWBR, this model appears beneficial in that it may support BRs increased autonomy and can balance collaboration equally between state and not state actors (Department of Environmental Affairs 2016; Pool-Stanvliet and Coetzer 2019).

Under the NGO model, the BR acts as a platform to bring together the interests of local stakeholders and is designed for collaboration, negotiating with other landscape actors as implementers of decisions made by the platform (UNESCO 2021). The CWBR’s BofD consists of individuals reflecting diverse stakeholder groups and institutions (UNESCO 2015; Hedden-Dunkhorst and Schmitt 2020) which is believed to be a good strategy for better cooperation amongst actors in the BR (Stoll-Kleemann and O’Riordan 2018), and avoids favouring narrow interests (Roldán et al. 2019). Doing so allows the CWBR to fulfill their collaboration and coordination role in the landscape – taking on the role of supporter and facilitator – as a *‘platform’* which brings together diverse stakeholders to deal with everyday challenges and aligning with UNESCO’s (1996) broader goals of fostering collaboration and coordinated efforts across BRs. As Müller (2014) and Stoll-Kleemann and O’Riordan (2018) describe the NGO model for BRs, the CWBR are not necessarily the implementers, but rather more often supporting actors in the landscape which are in need or will identify areas in need of action. This is a model in which many actors govern the SES and what Kooiman et al. (2008) and Edelenbos and van Meerkerk (2016) refer to as interactive governance. Interactive governance is important in this context as it can help deal with resource shortages and build resilience, i.e. the perceived lack of government support, human and financial resources, and helps to develop integrated responses and de-fragment actors in the landscape (Armitage et al. 2012; Müller 2014; Edelenbos and van Meerkerk 2016; Berkes 2017).

The CWBR is led by a BofD, which respondents acknowledge lacks gender and racial representation, although has recently begun to diversify through the addition of younger directors, and a female director—diversity is believed to increase board effectiveness (Bradshaw et al. 1998; Petrovic 2008; Ortega-Rodríguez et al. 2023). Directors do not have a fixed term of service, and a few have been serving the CWBR since its inception which benefits the BR in terms of its continuity and maintaining institutional memory. Skills, knowledge, and experience take time to replace, thus a consistent BofD with the addition of younger directors helps to transfer institutional knowledge, bring in new knowledge, ensure organizational memory, and can help mitigate corporate amnesia (Kransdorf 1998; Harvey 2012). Without the strategic addition of new directors (due to natural processes of staff turnover), the CWBR could however risk experiencing a static board in the long term in which new ideas, knowledge and perspectives are not incorporated.

Strategically selecting directors based on their expertise, knowledge, personal skills, and motivations for the company are important board qualities (Petrovic 2008). Directors are purposively selected depending on the skills needed, but also to prevent the possibility of board members *‘hijacking’* the organization by driving their personal agendas. Lessons from this model are to have a small board which offers an oversight role and is not involved in the implementation but rather connected to other organizations operating effectively in the landscape. Additionally, these need to be individuals which have an interest and are driven by some level of altruism given that participation on the board is voluntary.

There is significant overlap in the motivations of the board and their vision for the BR—described as *‘like-mindedness’*, and which is believed by participants to be a factor contributing to their effectiveness. Although this may increase the risk of groupthink (Petrovic 2008), a shared vision and limited internal conflicts have been found to increase the effectiveness of the board (Bradshaw et al. 2007; Ortega-Rodríguez et al. 2023).

Good meeting practices are considered essential for board effectiveness (Bradshaw et al. 1998; Ortega-Rodríguez et al. 2023). The CWBR have regular meetings. Board meetings, held quarterly, are described as a more informal setting, are a place to plan, discuss and vote on decisions. The BR management meet on a weekly basis which allows staff to be up-to-date and provide feedback on their programs. Importantly, ad hoc meetings and an *‘open phone policy’* between the CEO and BofD has developed a level of trust and allows decisions to be made quickly and without having to wait for the following meeting. This is consistent with Petrovic (2008) who states open and frequent communication contributes to building a shared vision and trust.

The structure of the CWBR is what Bradshaw et al. (1998) describes as an emergent model – characterized by networks, flexibility, and organic innovation. The CWBR structure grew organically and evolved to deal with new challenges, information, and requirements. However, this model requires strong and charismatic leadership (Bradshaw et al. 1998; George and Reed 2017; Stoll-Kleemann and O’Riordan 2018), and much of the BRs effectiveness and success is attributed to their operational leader, the CEO. However, the overreliance on one individual, the leader, comes with potential risk and consequences when the leadership changes.

Much like the NGO and emergent models which are *‘project-orientated’* and *‘issue-driven’* (Bradshaw et al. 1998; UNESCO 2021), the CWBR interacts with stakeholders and establishes partnerships on a *‘case-by-case basis’* much like its neighboring KBR (see Klaver et al. 2024) to address their most pressing needs. These stakeholders include a diverse group of provincial government departments, parastatals, NGOs, forums and working groups, local businesses, community organizations and sports clubs, while working particularly closely with local communities. This stakeholder engagement philosophy is to *‘find out who’s battling and help them to do better’* through support and facilitation, or to *‘fill the gaps’* or resource and capacity shortfalls within their local environment. CWBR frequently engage with local communities to co-create projects and solutions for challenges which they face. For example, ECD emerged as a need in the landscape. This agility to respond to challenges and create opportunities through partnerships enhances adaptive capacity (Lockwood et al. 2010). According to George and Reed (2017), collaborative partnerships are better equipped to develop and implement innovative solutions to problems by sharing knowledge and pooling resources. Although the assumption is that higher levels of participation, according to Arnstein’s (1969) ladder would increase BR legitimacy to greater degrees, in Roldán’s et al. (2019) survey across 92 BRs they found there is no linear relationship between participation and legitimacy, and that *any* levels of participation increase BR legitimacy amongst local stakeholders. Moreover, consistent engagement and dialog with stakeholders, as reported numerously in the Seville Strategy (UNESCO 1996), is fundamental to BRs as they are *“not only a designation or an international recognition*, [but] *a long-term commitment, a responsibility, a social, economic, and ecological project, that must be supported and carried out by its inhabitants”* (Bouamrane et al. 2016:6).

### Implementation Challenges in the Cape Winelands Biosphere Reserve

NPO governance and operation can be weakened by a lack of authority (Van Cuong et al. 2017). South African BRs have no *‘legal teeth’*, apart from the formally protected core zones, and are therefore implemented through *‘soft law’* (Pool-Stanvliet 2013; Pool-Stanvliet and Coetzer 2019), largely relying on adherence to national policy around linked matters of enforcement and/or management. However, social acceptance and support for the BR can be increased through stakeholder participation (Van Cuong et al. 2017) as well as effectiveness in producing outcomes (Lockwood et al. 2010) which may serve their recognition and coordination vision, and collaboration and coordination role.

Government involvement and commitment is critical for the BR success (Van Cuong et al. 2017). Although BRs are supported by national government during their nominations, South African BRs must largely find their own way to implement MAB (Pool-Stanvliet 2014). The CWBR, like Pool-Stanvliet (2013) found, lacks significant support from the national government and interest from local municipalities—noted by respondents as critically missing stakeholders, as opposed to better engagements with private stakeholders and communties. This is not particular to the CWBR case, and it has been found that in South Africa MAB faces challenges integrating vertically with national authorities and horizontally at the local level (Pool-Stanvliet 2014). Political buy-in is necessary for the long-term success of BRs (Coetzer et al. 2014), however the technical committee faced challenges in building reliable and consistent relationships between local municipalities and the CWBR. This is an important consideration for future action since the CWBR regards themself as a channel between the local communities and municipalities. Vhembe BR, for example, has established a number of *ex officio* non-voting director seats on their BofD (Hedden-Dunkhorst and Schmitt 2020). Doing similarly could result in more frequent engagement with the CWBR’s missing local government stakeholders—frequent engagement believed to improve stakeholder participation and support (Roldán et al. 2019).

Sustainable financial resources, or the lack thereof, can result in BR success or failure, and particularly a challenge for innovation, collaboration, and knowledge sharing (Van Cuong et al. 2017). The CWBR, like other South African BRs (Pool-Stanvliet and Coetzer 2020), lack financial resources, specifically operational funding, impacting on their ability to employ full-time staff. It is believed the CWBR could do much more if it were better resourced. Azadi et al. (2021) found that BRs in Africa receive very limited amounts of funding from government partly due to financial management systems hindering the process. This is the perception of the CWBR, which in some cases could not receive funding, for example to compile the ten-year Periodic Review (a UNESCO reporting requirement; UNESCO 1996), because of the mechanisms in place. Several lessons have emerged from this dilemma including building operational funds into project costs, service-level agreements for human resources and using overlaps with other actors in the landscape to pool resources. Taking advantage of the overlaps requires a shared vision and limited staff turnover as it could be detrimental to building/maintaining long-term relationships (Aunger et al. 2022). Although being under-resourced seems to foster innovation, human and financial resources are a necessity for continuity, competence and building trust (Stoll-Kleemann and O’Riordan 2018).

Awareness and communication are important factors determining the success or failure of BRs as it makes the BR concept a reality to those living within it and thereby enabling its implementation (Coetzer et al. 2014; Van Cuong et al. 2017). However, research elsewhere has indicated that BR inhabitants find the concept and its terminology difficult to understand (Pool-Stanvliet 2014). Social media is a useful way to support the dissemination of information, market the BR and build awareness (Coetzer et al. 2014; Van Cuong et al. 2017), which is a way in which the CWBR share news and project updates, and to encourage participation from their stakeholders – although it is believed that their interface with the public needs improvement. The CWBR is well-known in areas where their projects occur, however there are areas beyond, where the BR is still unknown. Increasing stakeholder enagement at any level, whether informing or delegating power (Arnstein 1969), is beneficial to BRs. Roldán et al. (2019) found BR stakeholders value receiving information in addition to providing inputs, especially project outcomes, which can result in better participation levels. Therefore, the CWBR would find it beneficial to continue their commitments to updating their local stakeholders regularly through various avenues—which is likely to have been key in gaining increased engagement and attendance at their stakeholder meetings.

Case study research is often criticized for non-generalizable findings (Bryman 2012). However, the aim of this research was to provide a contextualized understanding of how the CWBR interpret and implement global MAB policy within their local conditions. Thus, the sample was purposefully selected using specific criteria, which allowed an ‘information rich’ case to be built (Palinkas et al. 2015). The aim of this research was to fill the need for understanding the institutional context and governance strategies of BRs in the landscape in which they are found (Coetzer et al. 2014; Ferreira et al. 2018; Baldwin-Cantello et al. 2023; Barraclough et al. 2023). Despite these results not being directly transferable to other BRs, the need exists to understand and compare lessons learnt from BRs to improve the implementation success of the MAB (Coetzer et al. 2014; UNESCO 2017). A strength of the MAB, which is the absence of blueprints for implementation, offers an opportunity for place-based learning. Case studies such as this offer lessons and experience in implementing MAB. Future research should consider developing similar case studies in other BRs and *“to communicate the experiences and lessons learned, facilitating the global diffusion and application of these models”* (UNESCO 2017:52) to ultimately determine what works, for who, and why? Furthermore, research could additionally explore stakeholder perceptions and expectations of BRs to develop a holistic understanding of the BRs.

## Conclusion

The CWBR provided an interesting case as it has had to adapt to various iterations of MAB policy. This has meant that their implementation has been driven in response to emergent local needs and landscape priorities. As a result, any overlaps with the SDGs have largely been unintentional.

The CWBR strategy has been to focus on one core function at a time, become effective, achieve success, and thereafter expand efforts to other core functions. Their immediate priority had been their socio-economic development function. Targeting this function first may have alleviated pressures from external factors affecting their ability to successfully implement MAB. However, achieving success in this area has also resulted in increased legitimacy, ability to build a brand and attracted international partnerships which have been fundamental for the sustainability of the organization. An instrument in their success has been the type of people involved, strong and charismatic leadership and people willing to contribute to society through goodwill—a lifeline for the BR.

The CWBRs operational challenges—limited support, financial and human resources, and general awareness of the BR concept necessitate an NPO interactive governance model. This enables the CWBR to perform as a relational hub in the landscape relying on partnerships, building on overlaps, and interacting with, and de-fragmenting actors in the landscape to create opportunities for collective action across the BRs landscape actors.

There are no prescriptions for MAB implementation in individual BRs with this intentional flexibility providing an opportunity for place-based learning-by-doing for broader benefits. The research presented here contributes to a clearer understanding of how MAB is interpreted and implemented within a local African context. The place-based implementation of MAB allows the CWBR to evolve and adapt to local pressures. These in-depth learnings can aid MAB implementation elsewhere and provide insights in sustainability science. Lessons learned from the CWBR can be useful in other cases, and mechanisms used by the CWBR may be suitable for application for BRs operating in similar contexts, especially in the Western Cape of South Africa where four other BRs are located.

Available BR literature lacks in-depth knowledge from individual BRs and is skewed towards the Global North, leaving the Global South under-represented—specifically Africa. This research, although acknowledging the limitations of the chosen methodology, attempts to remedy the needs in BR literature by offering the CWBR as a Global South case study which shares knowledge and lessons of implementing the MAB. South Africa, although relatively new signatories to MAB, has been a part of the MAB network for more than two decades (Pool-Stanvliet and Coetzer 2020), and case studies such as this can offer valuable lessons in terms of practically implementing MAB in the country. The implementation of MAB could benefit from integrated comparative learning from in-depth BR case studies and therefore future research should follow suit to develop several globally representative ‘cases’ to share experiences and lessons learnt through the *learning-by-doing* approach of the MAB.

## Supplementary information

Supplementary Information

## Data Availability

Interview protocol available on request from K Coetzer (kaera.coetzer@up.ac.za). Links to data sources of the results can be found in Supplementary Table 1.

## Footnotes

1 CapeNature is the public entity responsible for conserving biodiversity within the Western Cape Province.

2 Refers to Section 21 of the Companies Act 61 of 1973 of South Africa and is a type of non-profit organisation.

3 At the time of this research, prior to the May 2024 National and Provincial elections producing no outright majority winner, South Africa’s national government and the Western Cape provincial government were led by opposition political parties.
