# Supplementary material for: Exploring the Implementation of UNESCO’s MAB Program in South Africa: A Case Study of the Cape Winelands Biosphere Reserve
Source: Environ Manage. 2024 Sep 18;74(6):1207–22. doi: 10.1007/s00267-024-02048-3 (PMC11549186; doi:10.1007/s00267-024-02048-3)
Supplement: Supplementary file 1 — Supplementary Information [file 267_2024_2048_MOESM1_ESM.pdf]

Supplementary Table 1: Source codes for all quotations of the results. Quotes incorporated into the narrative structure are written as normal. Verbatim quotes have beet *italicized*. Source codes include interview respondents (IR) and document results (DR).

Data	Source
The CWBR was initiated by the Cape Winelands District Municipality (CWDM) and the proposal put together by the Dennis Moss Partnership – a top-down establishment.	DR3, IR2, IR6
The CWBR was registered in 2007 after a four year long public participation process.	IR8
The impetus for its establishment was that it would be an important spatial development planning instrument and that development proposals would have to be approved by the committee.	IR3
This function is fulfilled by the Department of Environmental Affairs and Development Planning (DEADP), so to avoid duplication this role was never realized.	IR8
'damage control'	IR2
The CWBR *"didn't try to deal with all three* [BR core functions] *at a time. So* [the CWBR] *concentrate*[d] *on the education side"*	IR2
"[the CWBRs] *foci has been more community upliftment and education based*"	IR1
"The next side [the CWBR] *were weak, was the scientific side"*	IR2
The CWBRs projects are intended to align with the core functions of BRs as stipulated in The Statutory Framework of the World Network while responding to emergent local challenges.	IR3
"getting gender and racial representation on the board remains a challenge"	IR5
Each director is assigned to a portfolio, depending on their expertise, or a portfolio may be established for them.	IR1, IR7
It is the boards responsibility to provide an operational oversight role.	IR8
Linkages are present between the directors and some of the stakeholder groups to allow information to be shared and for fostering collaboration, for example with the CWDM, Heritage Committee, Iziko Museums, CapeNature and universities…	IR3, IR4, IR6, IR7, IR8
…the *"board's role,* [to] *a large extent, is to communicate and have that relationship with stakeholders"*	IR1
CWBR management consists of a chief executive officer (CEO), administrator and coordinator, and several project leaders, advisors or facilitators.	DR1, DR2
The CEO leads the management, oversees implementation, builds networks and relationships with funders and partners, and is tasked with timely decision-making.	IR1, IR3, IR4
The responsibility of the coordinator is reporting, managing records and administrative tasks.	IR2, IR3, IR4
Project leaders are appointed through service-level agreements and work part-time managing projects and facilitating various activities.	DR2
Directors are either sourced by or approach the CWBR to be elected to serve on the board.	IR2, IR7, IR8
Other criteria may include whether directors are involved in activities aligning with the CWBR objectives, and although not important at present, reside within the CWBR.	IR1, IR2
"the main thing is, what skills do [the CWBR] *need?"*	IR2
Directors are retained for as long as possible – until they are unwilling to serve or the lack of alignment between the objectives of the CWBR and what the director provides.	IR1, IR2
Whilst there are recent additions to the BofD, *i.e.* within the last 2 years, most of the directors have served for a minimum of five to seven years, but some directors have served since the establishment of the CWBR.	IR1, IR5, IR6, IR7
The CWBR has had a full-time coordinator since 2018.	IR1
'connecting with people'	IR1
'collaborative spirit' and '*leadership skills'*	IR7
'writing skills'	IR1, IR5
'objectivity'	IR4
'Built environment'	IR4
'environmental and *conservation ecology'*	IR3, IR6
'business'	IR2
'town and regional planning' and *'sustainable development'*	IR3, IR8
Spheres within government, public institutions, and the field of conservation	IR2, IR7, IR8
The CWBR has four full-time staff, including the CEO, coordinator, and project leaders whose work hours are flexible	DR2
These staff have specific responsibilities or commitments which need to be delivered upon.	IR1

This generally entails a 40-hour week but can be much longer – depending on what is required.	IR1
There are five to eight part-time workers depending on project funding, including administration, project leaders, advisors and facilitators.	DR2
Opportunities for training exist, both formal and informal, for example the coordinator completed a virtual BR management course.	IR1, IR2
Participants believe important full-time positions for the CWBR that should be filled include a CEO, coordinator, administrator, project leaders and a social media manager.	IR1, IR2
Board meetings, held quarterly, are a place for the BofD and CEO to plan, discuss and vote on decisions. Two thirds (4/6) of the BofD must be present for board meetings, including the secretary, chairperson and CEO, and all present need to vote.	IR1
Decision-making is perceived to be an informal process.	IR3, IR7
"the board meetings are a space to have conversations about things – to give input. But I wouldn't say there's a formalized decision-making structure ... I think it's quite loose".	IR5
Meeting agendas are shared beforehand allowing items to be attached.	IR5
Ad hoc meetings occur regularly, for example with municipalities.	IR1
'open phone policy'	IR1, IR2
There is trust between the CEO and directors and often decisions are made independently of the BofD and discussed at a later stage.	IR8
The CEO leads CWBR management meetings, a place to provide updates and work through programs, on a weekly basis. In these meetings there are usually 14 to 22 individuals depending on absentee numbers.	IR2
With regards to decision-making around CWBR projects, decisions are not made without the local communities. CWBR *"spend of lot of time in the communities"* attending community meetings through invitation where there is open dialogue in *'co-creating'* projects and solutions. Through these relationships being built, communities can regard the CWBR as a channel to the municipalities when they cannot get answers themselves, specifically regarding issues of housing, education and access to land.	IR2
There are no memberships, however the CWBR has a volunteer program.	IR4
There are generally six to eight volunteers (maximum of 14) who are either local or international.	IR1
Volunteers help with ongoing projects or initiate new projects depending on their skill set, for example the *'drone project'*.	IR1
The CEO often meets with the prospective volunteers beforehand in attempt to match the volunteers with specific projects or needs.	IR2
"BRs couldn't run as well as they do without volunteers and people being able to give of themselves"	IR1
The youth board consists of youth from different local communities…	IR8
"very powerful way of embedding [the CWBR] *in communities"*	IR2
It has been an experimental process and seen by some as relatively unsuccessful and therefore to be evolved into a youth committee or forum.	IR1, IR5
The advisory consists of individuals with knowledge on sustainable living and UNESCO-MAB.	DR1
The committee, which fulfils an advisory role to the board, consists of critical stakeholders, for example the local municipalities, CWDM, Department of Agriculture and CapeNature.	IR8
The committee meet once or twice a year and at the annual general meeting (AGM) to share knowledge and information, and feedback on activities.	IR6
"It's almost like stakeholder involvement... these are all the various stakeholders that are interested in the CWBR, and they need to be represented ... on the technical committee to make sure that they are happy with the direction [the CWBR] *is going. ..., most of the time the technical committee is made up of people that are coming from organizations that are already doing things. It should almost be for them to come along and tell us what they're doing. Then we can find out where we can help them a bit more, which does happen. But generally, those meetings, there's a lot of very quiet people there"*	IR6
'support and facilitate' actors in the landscape.	IR8
Participants perceive the CWBR as an organization to network, connect actors and to help those in need.	IR1, IR6
"[the CWBR] *will look at the problem. Diagnose it. What partners do* [the CWBR] *need to sort that out"*	IR2
Socio-economic development has been their focus because of the disparity in education in South Africa and the need to understand issues to solve them.	IR1, IR2
Their educational programs have been successful and gained them support.	IR2

There is an attempt to *'align with government departments'* and *'fill the gaps'* with their projects, for example early childhood development (ECD) a niche of the CWBR.	IR8
Their ECD program has put teachers through training and offered learning experiences with Iziko Museums.	IR7
The CWBR offer other forms of training for all interested parties, for example woodwork to upskill the local communities	IR2
The CWBR conducts environmental education and outreach programs with the youth and uses a mobile science unit (trailer) to promote conservation at schools.	IR2, IR3
The CWBRs conservation role is fulfilled through partnerships with several organizations, for example invasive alien plant (IAP) clearing with WWF and Idas Valley Trails, and with universities to fulfil their role in science and research.	IR1, IR2, IR5, IR6
The CWBR have developed strategic partnerships with several universities including, University of Stellenbosch, University of Cape Town (UCT) – specifically their African Climate Development Initiative, University of Leuven, and the Flemish Institute for Technological Research (VITO) in Belgium.	IR2
The CWBR support provincial government, WWF and CapeNature in research and monitoring through baseline data collection with the use of drones – a project initiated by a volunteer.	IR1, IR2, IR6
There are citizen science projects, for example their Source-to-Sea BeResilient project which focuses on conserving rivers and environmental education.	IR2
Lack of resources (*f*=7) is associated with the lack of funding, specifically operational funding which influences human resources and capacity.	IR3, IR6, IR7, IR8
The CWBR is believed to be over reliant on few human resources.	IR3, IR5
Partnering with other BRs to source funding seems to be an underutilized strategy.	IR2
"Funding is a major challenge. Most of the guys are doing it for the love of conservation"	IR8
"[The CWBR are] *still very reliant, heavily reliant on* [the CEO] *and* [the coordinator]. *There's a need to invest in human resources for the organization. Which comes along with funding questions"*	IR5
"There's scope for more, but it requires a larger dedicated team and operational funding"	IR7
"I don't understand why the Western Cape [BR] *Forum doesn't get together more. You know because the power of collaboration. I know it from business. I mean, there is big funding out there, but it's too big for one biosphere, but all 5 biospheres. We can go for the 50* [or] *100-million-europroject. But working in silos, we can't do that"*	IR2
Limited government support (*f*=4) uncovers a perceived lack of MAB program governance within South Africa, and therefore believed to be a lack of clarity in defined roles, structures and responsibilities within the *'chain of command'*, *i.e.* national MAB governance down to CWBR.	IR4, IR8
Participants believe national government are hesitant in allocating resources to the Western Cape for what is perceived to be political reasons.	IR2
There is the perception that their engagement is superficial.	IR6
It is believed there are structural challenges with regards to government involvement, for example, limited mechanisms to transfer funds, and believing that interacting with BRs is not part of their functional responsibility, *i.e.* no mandated engagements.	IR8
"It's frustrating that our system, our top-down structure – we don't have leadership on top"	IR8
"Again, who does the CWBR report to? What is our relationship? What are they doing? What is our contribution to helping them achieve their objective? Now for me that's not defined at all. We don't see any real government, either of provincial or national, participation really in terms of what to do. There's got to be a chain of command in all these organizations. Who are you? What are you doing? Why are you doing it? Who's effectively in charge of the whole thing? Power is what, we have no power. Things are devolved down to us to do, and to achieve what? To me those are not well defined, properly defined that people can understand or support for that matter. We have no support ... in real terms from national or provincial government"	IR4
"Treasury doesn't want money to come to the Western Cape. I mean, I've had that from [XXX]"	IR2
"[During technical committee meetings the CWBR] *get a 'representative' from the organization coming along. But not necessarily the right representative. It gets delegated down till somebody gets sent to you. That's not always useful"*	IR6

"I know they had challenges with the legislation ... they couldn't even transfer funds. I think 10,000 rand for reports. We compiled the report, a ten-year report. They said that that they will fund it, ... they couldn't fund it. They don't have a mechanism of transferring funds…"	IR8
"Guys at local government, if they have certain KPIs [key performance indicators]*, they focus on that. If the BR invites them, they just say, no, I'm not available… the person, individual at that organization must have similar interests to get them in"*	IR8
Awareness and communities (*f*=3) is associated with the awareness of the CWBR and the concept of BRs, getting people to understand issues which are not their immediate priority, as well as challenging community dynamics due the presence of gangsters.	IR2, IR3
Participants believe the current schooling system does not provide for many extracurricular activities which makes it challenging for the CWBR to engage with the youth.	IR6
"It's not really well known to the public, what we do, that we are there and we do all these things. I mean, the general public is not aware of us. Maybe in Franschhoek, with other projects there. People would know about it. In Stellenbosch, if you say BR. They say, what is that?"	IR3
"Getting communities to understand science, and climate change, and conservation is not easy.… hungry stomachs have no ears"	IR2
"We've got a difficult biosphere in that a lot of the community is gang controlled"	IR2
"It's very, very difficult to get to the kids, which I think is probably one of the most important things. Especially the way things are going now. If you don't have any understanding of the natural world, you're going to have absolutely no desire to do anything about it"	IR6
Participants suggested it is the continued perseverance and voluntary commitment of the team.	IR1, IR7
Other factors include the leadership, like-mindedness, personalities and networks within the team involved.	IR1, IR7
Participants attributed much of the success of the CWBR to the CEO.	IR3, IR4, IR6
"If it wasn't for [the CEO]*, none of this would have happened. None of it would happen.* [The CEO is] *a rather phenomenal person"*	IR6
Some credited the partnership between the CEO and coordinator together with the relaxed and flexible nature of engaging with the team.	IR2, IR3
When asked about instrumental positions which have increased the CWBRs effectiveness, one participant believes success has come from the team's passion and commitment to meaningful work	IR8
while another explained that it is more the types of people and personalities involved and not necessarily their position (see below).	IR6
"The structure has got nothing to do with it. If you don't have the right people there. Or if you do have the right people, you can have any structure, the structure isn't the issue. It's the personalities and whether they [are] *prepared to do what they're supposed to do or not, you know. You can have the best structure in the world and a bunch of 'palookas' sitting in it and it's still not going to work."*	IR6
Stakeholder participation occurs *"on a case-by-case basis"*...	IR5
…and could include any actors in the CWBR of which CWBR stakeholder mapping has identified for example provincial government departments, non-governmental organizations, community forums and water catchment area working groups, local businesses, community organizations and sports clubs.	DR2
One participant believes partnerships cannot be forced and that one must attract them by providing value, which the CWBR have done in their educational domain.	IR8
Participation from all interested and affected parties is encouraged through blog posts on their website, newsletters, word-of-mouth, and via phone call.	IR5, IR7
Participation levels are increasing – several participants said that they have had increasing attendance at their AGMs.	IR4, IR7
Participants reported challenges with regards to stakeholder participation from local government: there is superficial engagement and lack of perceived significance of the CWBR.	IR6, IR8
Furthermore, some stakeholders are perceived to be prioritizing their own agendas…	IR7
… or that the CWBR does not feature on the stakeholder's agenda, or within their professional *'functional responsibility'* unless the individual has a personal interest.	IR8
Stakeholder participation from local and national government (*f*=4) is believed to be missing despite being considered critical partners.	IR2

Participants believed that municipalities are disinclined to play an active role in CWBR engagement/participation, with room for more engagement.	IR2, IR8
Furthermore, participation from some key conservation bodies (*f*=2) are believed to be missing. The absence of these stakeholders is a *'limitation'* for the CWBR.	IR3
The CWBR would like more participation from the public (*f*=2) particularly youth groups.	IR1
Institutional overlap occurs with other actors in the landscape in terms of their mandate, for example CapeNature and municipalities.	IR2, IR6
Participants believe the challenge therein is to reduce duplication, and resource competition.	IR1, IR2, IR7, IR8
Participants mentioned the opportunity is to form partnerships, pool resources and collaborate – *'dovetailing'* projects with CapeNature, for example.	IR2, IR4, IR6
Participants were unsure whether such overlap enables or constrains ongoing/further government support. Participants believe it depends on who is involved as one needs a shared vision and the *'collaborative spirit'*,	IR7
…while another said that it could enable support,…	IR1
however staff turnover is a challenge when it comes to building long-standing relationships.	IR6
Participants perceive some municipal councilors to be disruptive in their attempts to politicize the CWBR…	IR2
…and that even directors have the potential to be disruptive by trying to push their agendas and in some way *'hijack'* the CWBR.	IR8
Furthermore, some government departments are believed to be disruptive and of little help to the CWBR – in some cases perpetuating and exacerbating issues, for example land invasions.	IR4
"get a nice core team together"	IR3, IR7
…a good CEO or leader.	IR3, IR4
a physical space is beneficial to have,	IR5
setting a clear plan (strategy or business plan) early on,	IR2
to find a niche…	IR8
and if there are overlaps with other actors look to build partnerships and collaborate.	IR1
Partnerships and continuity of funding are considered the *'lifeline'* of a BR (IR5).	IR5
A participant advised to try form these early and develop the BR around the personalities involved (IR8).	IR8
"Some active individuals who are enthusiastic and make their time available to their own societies… I think the important thing is to identify two or three people who are really committed and prepared to put in time."	IR3
"Get yourself a good CEO."	IR3
"You need a charismatic person that's willing to take on the overall role. Then for him or her using your own personal charisma to persuade other people to join this enterprise."	IR4
"I think having the [physical] *space* [headquarters], *where volunteers ... can stay. There's an office ... Everything is in one place… and I think that's valuable... it's really the ideal scenario. You've got someone who is setting up and championing something like this. If they have ... the physical space that they're working in, that can become the HQ... It just makes things a lot easier."*	IR5
"Find your niche, ... [where] *you can have an impact. It's about just searching for that, networking, understanding the landscape."*	IR8
"I think having ... stability of funding in place, I think there's something linked to that around kind of international partnerships and relationships. I think that's something which [the CEO] *has done quite well. It's really been a lifeline to the organization, in many ways. I think ... for BRs in the Global South, establishing partnerships and relationships with either funders and/or other BRs in the* [Global] *North as a way of trying to kind of create some sense of financial stability."*	IR5
"... the [BR] *is dependent on funding and the personalities that drive it. If the personalities that drives the BR, if they do not conform to a rigid system, a rigid structure. If it's easier for them to implement without that structure, then I just think it should go with the flow. It shouldn't be overregulated in terms of* [a] *set structure."*	IR8
